# Genomic and Epigenomic Insights into Nutrition and Brain Disorders

**DOI:** 10.3390/nu5030887

**Published:** 2013-03-15

**Authors:** Margaret Joy Dauncey

**Affiliations:** Wolfson College, University of Cambridge, Barton Road, Cambridge, CB3 9BB, UK; E-Mail: mjd4@cam.ac.uk; Tel./Fax: +44-1223-335908.

**Keywords:** Alzheimer’s disease, genomics, epigenomics, non-coding RNAs, neurodevelopment, neuropsychiatry, neuroscience, nutrition, Parkinson’s disease, schizophrenia

## Abstract

Considerable evidence links many neuropsychiatric, neurodevelopmental and neurodegenerative disorders with multiple complex interactions between genetics and environmental factors such as nutrition. Mental health problems, autism, eating disorders, Alzheimer’s disease, schizophrenia, Parkinson’s disease and brain tumours are related to individual variability in numerous protein-coding and non-coding regions of the genome. However, genotype does not necessarily determine neurological phenotype because the epigenome modulates gene expression in response to endogenous and exogenous regulators, throughout the life-cycle. Studies using both genome-wide analysis of multiple genes and comprehensive analysis of specific genes are providing new insights into genetic and epigenetic mechanisms underlying nutrition and neuroscience. This review provides a critical evaluation of the following related areas: (1) recent advances in genomic and epigenomic technologies, and their relevance to brain disorders; (2) the emerging role of non-coding RNAs as key regulators of transcription, epigenetic processes and gene silencing; (3) novel approaches to nutrition, epigenetics and neuroscience; (4) gene-environment interactions, especially in the serotonergic system, as a paradigm of the multiple signalling pathways affected in neuropsychiatric and neurological disorders. Current and future advances in these four areas should contribute significantly to the prevention, amelioration and treatment of multiple devastating brain disorders.

## 1. Introduction

Advances in genomics and epigenomics are revolutionizing understanding of mechanisms underlying brain disorders [1,2,3,4]. Considerable evidence suggests that many neuropsychiatric, neurodevelopmental and neurodegenerative disorders are linked with multiple complex interactions between genetic factors and environmental variables, such as nutrition [5,6,7]. Indeed, numerous diets, foods and nutrients are implicated in optimal and sub-optimal brain health throughout the life-cycle [8,9,10,11,12,13,14,15,16].

Nutrition affects multiple aspects of neuroscience including neurodevelopment, neurogenesis and functions of neurons, synapses and neural networks in specific brain regions [17]. Nutrition-gene interactions play a critical role in these responses, leading in turn to major effects on brain health, dysfunction and disease [5,6,7,18]. Individual differences in multiple gene variants, including mutations, single nucleotide polymorphisms (SNPs) and copy number variants (CNVs), significantly modify the effects of nutrition on gene expression. A further layer of regulation is added by differences in the epigenome, and nutrition is one of many epigenetic regulators that can modify gene expression without changes in DNA sequence.

Epigenetic mechanisms play a major role in developmental, physiological and pathological processes [19,20]. They include DNA methylation and hydroxymethylation, histone modifications and higher order chromatin remodelling, and non-coding RNA (ncRNA) regulation. Activation of these mechanisms affects the expression of multiple genes involved in cell development, signalling and function [4,5,6,7,21,22,23]. Numerous epigenetic regulators, including environmental factors, interact with multiple gene variants to produce striking individual differences in responses to nutrition. Critical interactions also exist between specific dietary components, and between nutrition and other environmental variables such as stress, infections, social interactions, season of birth and stage of development [5,6,7,24,25,26,27]. These epigenetic regulators, together with multiple genetic factors, comprise a sophisticated control network that plays a central role in brain function.

**Figure 1:**
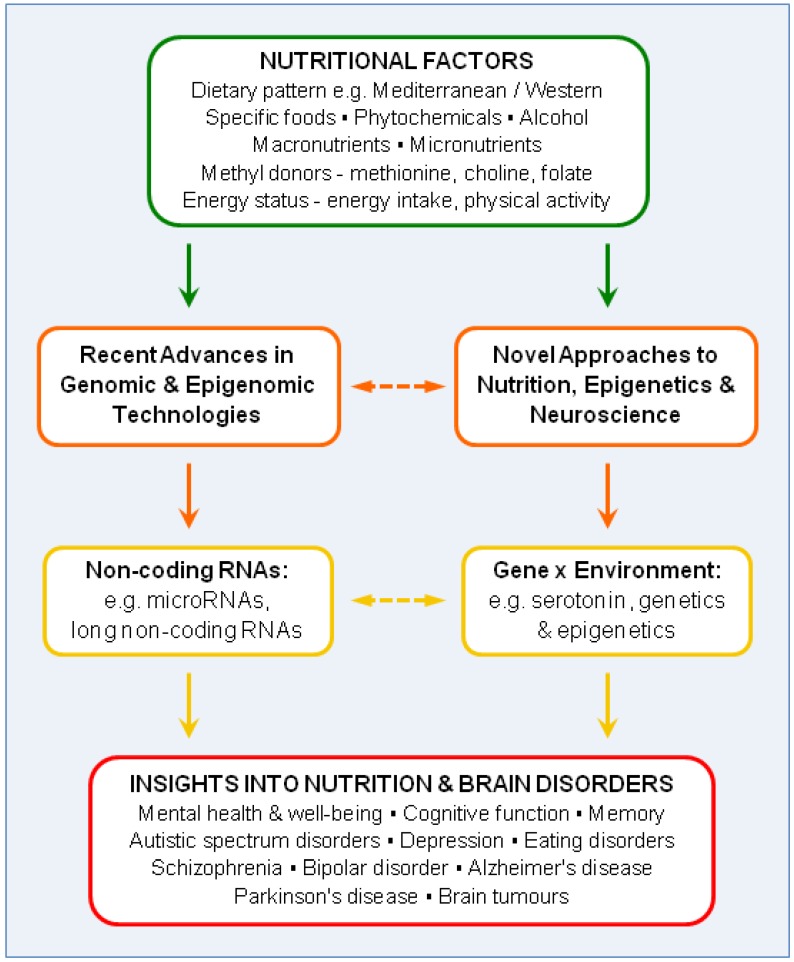
Nutrition-gene interactions and brain disorders: Outline of current review.

The current review highlights the significance of advances in genomics and epigenomics to an understanding of the role of nutrition in brain disorders. Studies using genome-wide analysis of multiple genes and comprehensive analysis of specific genes are together elucidating mechanisms underlying responses of the brain to nutrition. This is a vast field of research, involving many hundreds of publications every month. The present review therefore focuses on recent progress in four key areas (Figure 1): First, a short overview is given of significant advances in genomic and epigenomic technologies over the last few years. These are revolutionizing our ability to evaluate mechanisms underlying neurological responses to nutrition, and hence to determine outcomes in relation to health or disease. Second, to illustrate the potential of these technological advances, the role of ncRNAs, and especially microRNAs (miRNAs) and long non-coding RNAs (lncRNAs), is addressed because of their emergence as critical regulators of transcription, epigenetic processes and gene silencing. Third, novel approaches to nutrition, epigenetics and neuroscience are discussed, especially in relation to the role of exogenous factors in determining neurological phenotype and disorders. Finally, the relevance of these novel approaches is addressed in relation to gene-environment interactions in the serotonergic system, as an example of just one of the multiple neural signalling pathways that are affected in neuropsychiatric and neurological disorders. Current and future advances in these four areas should contribute significantly to the prevention and treatment of multiple devastating brain disorders.

## 2. Recent Advances in Genomic and Epigenomic Technologies

During the last few years, major advances in DNA sequencing technology have revolutionized understanding of the mechanisms underlying complex biological problems and multifactorial diseases [28,29]. Compared with methods used by the Human Genome Project [30], modern sequencers are 50,000-fold faster and have dramatically reduced the cost of DNA sequencing by a factor of more than 50,000. These new technologies have thus enabled major advances in understanding of human genomics and epigenomics. Especially significant are new insights into individual genetic variability, and the critical role of non-coding RNAs (ncRNAs) in epigenetics and gene regulation.

### 2.1. Whole Genome Strategies, Next-Generation DNA Sequencing and Brain Disorders

Successful and early completion of the human genome sequence in 2001 was based on the classic electrophoretic Sanger sequencing method [30]. However, this method was unsuitable for studying genetic variability. Instead, individual genotypes were assessed using specific DNA sequence probes targeted at known SNP positions. At this time, it could not have been predicted that next-generation sequencing technology would enable the complete analysis of all the functional elements and variations in the human genome. By contrast with the limited scalability of the Sanger method, this new approach uses image-based massively parallel sequencing-by-synthesis platforms. Together with a marked increase in analytical throughput, this has catapulted genome-sequencing into a multi-purpose tool for mapping epigenetic modifications of the genome, and the complete assessment of protein-coding and non-coding RNA transcripts. In the longer term, further advances in data assessment may enable complete analysis of the relation between genomic variation and phenotype.

Over the past five years, new technologies have provided an overview of both common and rare genetic variability across the whole genome that has significantly improved understanding of many brain disorders [1,2]. These include Alzheimer’s disease, schizophrenia, bipolar disorder, major depressive disorder, autistic spectrum disorders, attention-deficit hyperactivity disorder, anorexia nervosa, alcohol dependence and nicotine dependence. Data on structural variants, rare exonic variants, and an increasing number of common variants have helped to identify risk factors for the development of several complex disorders. Moreover, they support novel hypotheses related, for example, to (a) cholesterol metabolism and the innate immune response in Alzheimer’s disease, (b) a network involving the microRNA miR-137 in schizophrenia, (c) calcium signalling in bipolar disorder and schizophrenia, and (d) chromatin remodelling in autism.

Genome-wide association studies (GWAS) involve many thousands of patients and control subjects, to overcome problems associated with the huge number of interrogated genetic variants. This approach has been used with considerable success to identify many disease-predisposing variants. For example, recent studies have identified: a low-frequency variant in the amyloid-β precursor protein (*APP*) that protects against Alzheimer’s disease and age-related cognitive decline [31], a common variant conferring risk of psychosis [32], common SNPs and rare copy number variants (CNVs) that may confer risk to anorexia nervosa [33], and susceptibility loci for the most common form of migraine, a disabling episodic neurovascular brain disorder affecting 12% of the general population [34]. Despite these significant advances, it is now appreciated that future studies aimed at identifying disease-associated low-frequency and rare variants may need to involve even larger sample sizes than those currently used in GWAS, in order to achieve the necessary statistical power [35,36]. This approach should prove especially rewarding in the study of many complex brain disorders that result from both genetic and environmental factors, including Alzheimer’s disease, autism, schizophrenia and eating disorders. Moreover, when complemented by a systems genetics approach the outcome will be particularly informative. Systems genetics is a specialized version of systems biology that seeks to reveal multiple complex connections from genetic variation, through intermediate phenotypes such as gene coexpression networks, to overlying systems level phenotypes [37,38,39,40]. The importance of an integrative approach to understanding the mechanisms linking genetic variability, nutrition-gene interactions and phenotypic diversity is illustrated in Section 5 of this review.

Current knowledge suggests that assessing multiple forms of genetic variation is likely to yield many new findings in neuroscience. The challenge for nutritionists is to coordinate these findings with novel approaches to assessing the role of interactions between environmental factors and genotype. Recent findings from two key projects that are currently providing new understanding of genetic variability and epigenetic mechanisms, The 1000 Genomes Project and ENCODE, are therefore discussed in the following paragraphs.

### 2.2. Genetic Variation and the 1000 Genomes Project

Genetic variability underlies not only individual differences in responses to nutrition but also the propensity of individuals for specific brain disorders. Major advances in gene sequencing technology have enabled characterization of the vast majority of human SNPs and many structural variants, including CNVs, across the human genome. The 1000 Genomes Project is an international sequencing collaboration launched in 2008 to produce an extensive catalogue of all types of human genetic variation. The project published pilot data in 2010 [41], and will have sequenced the genomes of approximately 2500 people from 25 global populations when complete.

The three studies in the pilot phase involved low-coverage whole-genome sequencing of 179 people from four populations, high-coverage sequencing of two mother-father-child trios, and exon-targeted sequencing of 697 people from seven populations [41]. The results described the location, allele frequency and local haplotype structure of approximately 15 million SNPs, 1 million short insertions and deletions, and 20,000 structural variants, most of which had not been described previously. Each person was found to carry approximately 250–300 loss-of-function variants in annotated genes, and 50–100 variants previously implicated in inherited disorders.

More than 95% of common (>5% frequency) variants were discovered in the pilot phase of the 1000 Genomes Project, thus paving the way for investigation of the links between genotype and phenotype. However, lower-frequency variants, especially those outside the exome, remained poorly characterized. Nevertheless, it was appreciated that low-frequency variants are enriched for potentially functional mutations, and that characterizing such variants, for both point mutations and structural changes, was likely to identify many functionally important variants and be crucial for interpreting individual genome sequences. Highly significant progress has recently been made in this area.

In November 2012, following extensive genomic assessment of 1092 people from 14 populations world-wide, an integrated map of human genetic variation was published [42]. This provides an exceptional resource describing human genomic variability. It encompasses a haplotype map of 38 million SNPs, 1.4 million short insertions and deletions, and more than 14,000 larger deletions. Profiles of rare and common variants differ between populations, and low-frequency variants show substantial geographic differentiation. Moreover, each person contains hundreds of rare non-coding variants at conserved sites, such as motif-disrupting changes in transcription factor binding site. The considerable significance of these non-coding variants to brain disorders is discussed in Section 3 of the current review.

### 2.3. ENCODE: An Encyclopedia of DNA Elements in the Human Genome

A series of articles by ENCODE, the **Enc**yclopedia **o**f **D**NA **E**lements, in September 2012 has critical implications for providing insights into the organization and regulation of our genes and genome [3]. ENCODE is an international research consortium launched in 2003 and funded by the National Human Genome Research Institute to identify all functional elements in the human genome sequence, *i.e.*, all regions of transcription, transcription factor association, chromatin structure and histone modification. Their data now enable biochemical functions to be assigned to 80% of the genome, in particular outside the well-studied protein-coding regions.

ENCODE’s massive enterprise has involved the production and initial analysis of 1640 data sets, and integration of results from studies involving 147 cell types and data from other resources including genome-wide association studies (GWAS). Key findings indicate that (a) most of the human genome takes part in at least one biochemical RNA- and/or chromatin-associated event in at least one cell type; (b) classifying the genome into seven chromatin states indicates an initial set of 399,124 regions with enhancer-like features and 70,292 regions with promoter-like features; (c) promoter functionality can explain most of the variation in RNA expression; (d) the number of non-coding variants in individual genome sequences is at least as large as those within protein-coding regions; (e) SNPs associated with disease are enriched with non-coding functional elements, and in many cases the disease phenotypes are associated with a specific cell type or transcription factor. 

The significance of these findings is immense and of profound relevance to future investigations on the role of nutrition-gene interactions in health and disease. In the words of the head of the team working on the GENCODE sub-project [43,44]: “If the Human Genome Project was the baseline for genetics, ENCODE is the baseline for biology, and GENCODE are the parts that make the human biological machine work. Our list is essential to all those who would fix the human machine.”

### 2.4. Epigenomics and New Technologies

Approximately 70% of all abstracts featuring “epigenome” have been published within the last three years [45]. This proliferation of investigations on epigenomics, *i.e.*, the dynamic regulatory layers that modulate expression of the genome’s static DNA sequence, has been possible because of major advances in high-throughput technologies. These enable the analysis and positioning of epigenetic markers such as DNA methylation, histone modifications and chromatin states within the biological context of the whole genome.

Epigenome core technologies are now largely standardized, although advances in sensitivity and resolution are still being made [46]. The standard assay for transcription factor binding, and mapping genome-wide distribution of histone modifications, is chromatin immunoprecipitation followed by sequencing (ChIP-seq). Similarly, techniques for DNA methylation use methylated DNA immunoprecipitation, to enrich the methylated DNA sequences, followed by high-throughput sequencing (MeDIP-seq). Methods are now tending to converge on whole genome bisulphite sequencing because of its high resolution combined with reduced costs. The desire to map the entire methylome is driving further developments in large-scale DNA methylation profiling methods. Comparison of MeDIP-seq with the targeted approach of the Infinium HumanMethylation450 BeadChip has revealed strengths and weaknesses in both methods [47]. In particular, the former technique allows the detection of almost twice as many differentially methylated regions as the latter, including thousands of non-RefSeq genes and repetitive elements that may be important in disease. 

Data from epigenome studies are providing information of far greater significance than simply mapping specific epigenetic marks to a given cell type. Key applications are related to genome annotation, cell identity and disease [46]. As the ENCODE project has demonstrated [3], investigations based on the primary DNA sequence alone cannot provide a comprehensive understanding of the mammalian genome. Chromatin signatures, for example, enable efficient and precise genome annotation of regulatory elements and can locate functional or cell type-specific regions of interest. Epigenomic maps provide more information than is gained from gene expression data alone. Precise chromatin states can clarify a gene’s activity status, which in turn has consequences for how specific gene loci behave in normal development and disease. Moreover, epigenomic maps are playing an important role in studies of brain disorders, and are clarifying affected pathways and identifying novel prognostic methylation biomarkers, for example in neuroblastomas [48,49].

**Figure 2:**
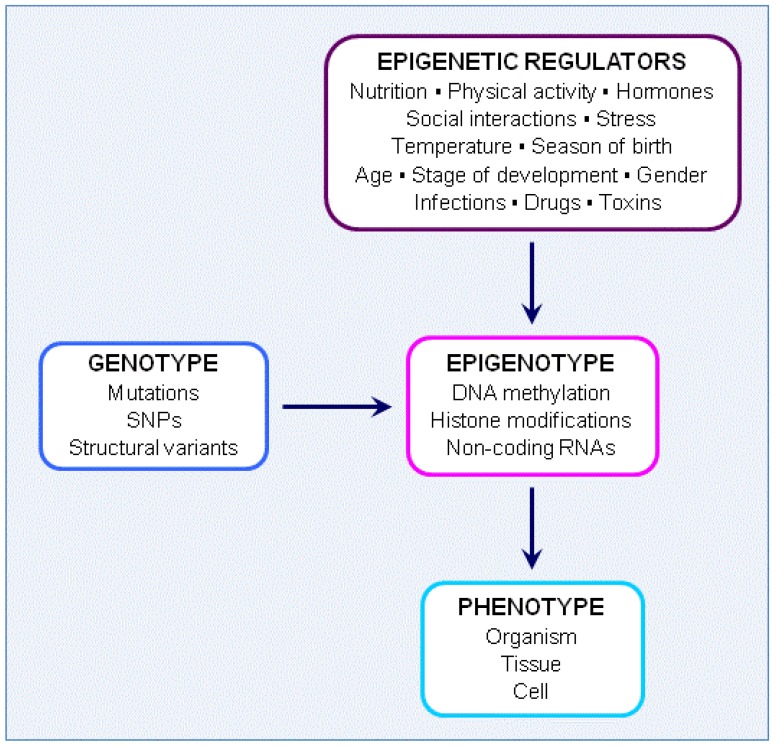
Interactions between genotype, epigenotype and phenotype.

Especially powerful is the combination of epigenome-wide association studies with genome-wide association studies (GWAS) to locate disease-relevant regulatory elements [3,50]. This combined approach will prove especially valuable for providing further insights into the multiple roles of nutrition in brain development and function. The relevance of combining studies on genotype and epigenotype is highlighted in Figure 2 and is discussed further in the following sections.

## 3. Non-Coding RNAs (ncRNAs), Gene Regulation and Neuroscience

The Human Genome Project provided ground-breaking information on the human genome sequence [30], and this was followed by considerable advances in identifying protein-coding genes. However, understanding of the genome, especially with respect to ncRNAs, alternatively spliced transcripts and regulatory sequences remained far from complete. Indeed, since only 2.2% of human genomic DNA is protein-coding, the rest was sometimes termed “junk DNA” [51]. The possibility existed, however, that these non-coding regions may have a functional role in complex regulatory processes, thus explaining the relatively low number of protein-coding genes in humans, an evolutionarily advanced species. Significant advances in the last few years have shown that this is indeed the case.

### 3.1. Genomic and Epigenomic Regulation by ncRNAs

During the decade following publication of the human genome sequence, the centrally held paradigm of gene expression, that DNA is transcribed into messenger RNA (mRNA) which is then translated into protein, has been gradually undermined. Advances in new technologies have enabled the unexpected discovery of multiple classes of RNAs that are not translated into protein, *i.e.*, non-coding RNAs (ncRNAs), and yet play key roles in transcription, epigenetics and gene function [3,52,53]. The complexity of gene regulation is further revealed by the discovery that most of the genome is transcribed in both the sense and antisense directions. Natural antisense transcripts (NATs) are transcribed from the opposite strand of protein-coding and non-protein-coding genes and act as epigenetic regulators of chromatin remodelling and gene expression [54].

The multiple types of ncRNA are often classified according to size as small or long (Table 1). Several years ago, the role of small ncRNAs in developmental gene regulation was discovered but only recently has the significance of the abundant and versatile long ncRNAs (lncRNAs) been appreciated. They have a broad range of functions, including roles in transcriptional and epigenetic mechanisms via recruitment of transcription factors and chromatin-modifying complexes to specific nuclear and genomics sites, post-translational RNA modifications, nuclear-cytoplasmic shuttling, and translational control.

**Table 1:** Examples of non-coding RNAs (ncRNAs) and their molecular functions (for further details, see [55,56]).

**Small ncRNAs** (<200/400 nucleotides)
*MicroRNAs* (*miRNAs*): Mainly induce translational repression; involved in post-transcriptional gene silencing, or deadenylation and degradation *Endogenous small interfering RNAs* (*endo-siRNAs*): Induce degradation or heterochromatin formation *PIWI-interacting RNAs* (*piRNA*): Epigenetic and possibly translational control via complementarity with DNA or RNA sequences *Transcription initiation RNAs* (*tiRNAs*): Possibly promote transcription via epigenetic regulation
**Long ncRNAs **(>200/400 nucleotides, sometimes >100,000 nucleotides)
*Long intergenic ncRNAs* (*lincRNAs*): Epigenetic regulation *Natural antisense transcripts* (*NATs*): mRNA transcription, splicing, stability and translation; epigenetic modifications; precursors of endo-siRNAs *Expressed non-coding regions* (*ENORs*): Transcriptional regulation, genomic imprinting, precursors of other short and long ncRNAs *Enhancer RNAs* (*eRNAs*): Transcriptional regulation

### 3.2. NcRNAs and Brain Disorders

Dramatic progress in RNA biology over recent years is of particular significance to brain disorders because neurons are highly transcriptionally active and demonstrate strong expression of ncRNAs. Indeed, many ncRNAs play a vital role in normal brain function, and are involved in neural development, plasticity, memory and cognition [55,56,57]. Epigenetic processes are often involved, suggesting key interactions between ncRNAs and environmental factors such as nutrition. 

Initial studies on the relevance of ncRNAs to disease tended to focus on the disruption of miRNA expression in cancer. These have provided new insights into the molecular basis of disease and suggested novel approaches to investigation of brain disorders, including brain tumours. In the central nervous system, balanced expression of miRNAs plays an important role in preventing neurodegeneration, and misregulation of miRNA pathways appears to be essential in the pathogenesis of several age-dependent neurodegenerative disorders [58]. Indeed, dysregulation of numerous types of ncRNA is linked with many diseases [59,60], and especially significant is evidence that ncRNAs are involved in the pathophysiology of every major class of neurological and neuropsychiatric disorder [55,56,61].

Considerable evidence suggests central roles for ncRNAs in Alzheimer’s disease. Two of the most important proteins linked with Alzheimer’s, amyloid precursor protein and BACE-1, are extensively controlled by multiple miRNAs, NATs and other ncRNAs [56,62]. Alzheimer’s disease brains are deficient in brain-derived neurotrophic factor (BDNF), a key regulator of synaptic plasticity and memory, and of major significance is the recent finding that miRNA-dependent dysregulation of BDNF participates in the pathogenesis of Alzheimer’s [63]. In postmortem brain samples from a mouse model and humans with Alzheimer’s, miR-206 is upregulated. The concomitant decrease in BDNF expression is restored by the antagomir AM206, a neutralizing inhibitor of miR-206. Moreover, the increase in brain BDNF levels with AM206 enhances hippocampal synatophysin expression and neurogenesis in the mouse model, suggesting that it can improve cognitive function. The effects of food intake and physical activity on brain health are mediated in part via BDNF [5,6,7,8,64]. This suggests the possibility that miR-206 plays a role not only in Alzheimer’s but also in mediating energy status-BDNF interactions in healthy individuals. The relevance of these findings is discussed further in Section 5 of this review.

Evidence also links miRNAs with the second most prevalent neurodegenerative disorder, Parkinson’s disease [56,65]. For example, a miRNA pathway regulates dopaminergic neuron development and function, and expression of α-synuclein, a protein linked with Parkinson’s pathology, is affected by several miRNAs. Especially important is the finding that genetic polymorphisms of miRNA related sequences confers risk for Parkinson’s disease.

The risk of brain disorders is influenced by genetic variation not only in protein-coding genes but also in ncRNAs and their targets. In Parkinson’s disease, a SNP in the 3′ untranslated region of the fibroblast growth factor 20 gene (*FGF20*) disrupts a binding site for miR-433, increasing translation of *FGF20* [66]. This increase is correlated with increased α-synuclein, which can lead to Parkinson’s disease. Moreover, miRNAs contribute to retinoic acid induced differentiation in neuroblastoma [67]: miR-10a and miR-10b play a key role in neural cell differentiation through direct targeting of nuclear receptor corepressor 2 and concomitant downregulation of MYCN, a potent oncoprotein in neuroblastoma. Epigenetic deregulation of many ncRNA genes is also associated with brain disorders. Large-scale cancer genomics projects are profiling hundreds of tumours at multiple molecular layers including miRNA expression, with the long-term possibility of developing small RNA therapeutics specific for subtype or individual [68]. For example, overexpression in neurospheres of two predicted proneural drivers, miR-124 and miR-132, leads to partial reversal of tumour expression changes. 

### 3.3. NcRNAs, Nutrition and the Brain

**Figure 3:**
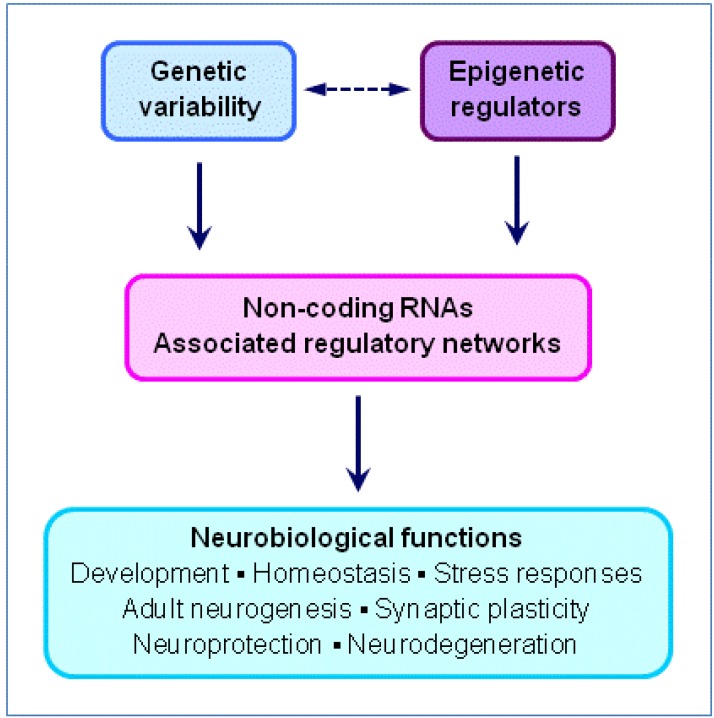
Regulation and neurobiological functions of non-coding RNAs.

The non-coding transcriptome has extraordinary functional diversity and environmental responsiveness, and the expression of ncRNAs is controlled by numerous transcriptional and epigenetic factors, including nutrition. NcRNAs, together with their associated regulatory networks, in turn have critical influences on the brain via their multiple neurobiological functions (Figure 3).

In recent years there has been increasing interest in nutritional modulation of miRNAs, throughout the life cycle. Significant advances have been made in relation to nutritional regulation of miRNAs in cancer [69]. Dietary factors including folate, choline, retinoic acid, vitamin D, vitamin E, selenium, omega-3 fatty acids, butyrate, phytochemicals and resveratrol modify miRNA expression and their mRNA targets in several cancer processes. These include apoptosis, cell cycle regulation, differentiation, inflammation, angiogenesis, metastasis and stress response pathways. The extent to which nutrition-gene interactions may modulate miRNAs associated with risk of brain tumours remains to be established. 

Polyphenols are the most abundant antioxidants in the human diet and occur widely in fruits, tea, coffee, cocoa and red wine [9]. Epidemiological, clinical and animal studies suggest a role for polyphenols in cognitive function and neurodegenerative disorders [12,70]. Gene variants of the apolipoprotein apoE are linked with risk of Alzheimer’s: apoE4 increases risk whereas apoE2 may protect against Alzheimer’s [5,6,7]. Of particular interest, therefore, is the recent finding that dietary polyphenols modulate hepatic miRNA expression in apoE deficient mice [71]. Polyphenols appear to counteract the modulation of miRNAs induced by knock-out of the *apoE* gene. Five of the differentially expressed miRNAs were regulated in common by all nine of the different polyphenols investigated, suggesting a common mode of action for polyphenols. Precise functions and roles of these miRNAs, and the extent to which the apoE genotype may modulate the action of polyphenols on miRNA expression remain to be investigated. However, taken together, these studies suggest that dietary polyphenols exert beneficial effects on brain function by influencing cellular energy metabolism, miRNAs and signalling pathways of key molecules in neural plasticity such as BDNF.

Much remains to be discovered about the nutritional modulation of ncRNAs, especially in relation to cell-specific responses and interactions with other epigenetic regulators. Physical activity, for example, plays a key role in regulating miRNAs and DNA methylation [72,73,74]: following acute exhaustive exercise, there are unique and dynamic alterations in circulating-miRNAs, suggesting that they may serve important endocrine functions. Moreover, future studies on the role of nutrition in regulating long non-coding RNAs (lncRNAs) may be especially beneficial to understanding of mechanisms underlying brain disorders. 

## 4. Novel Approaches to Nutrition, Epigenetics and Neuroscience

Serendipity plays a significant role in advancing scientific knowledge and an open, informed mind is essential for optimal progress in the biomedical sciences. Concomitant with progress in genomic and epigenomic technologies, significant advances are being made using novel approaches to the study of nutrition-gene interactions and neuroscience. 

The development, homeostasis and plasticity of the central nervous system are mediated by epigenetic mechanisms that regulate gene expression, without changes in DNA sequence, in response to endogenous and exogenous environmental variables. Substantial evidence also implicates epigenetic mechanisms in numerous neuropsychiatric and neurological disorders including schizophrenia, bipolar disorders, Alzheimer’s disease, Parkinson’s disease and primary brain tumours [4,5,6,7,20,75,76].

Nutrition has a major influence on the epigenome although its precise role is difficult to establish because of multiple interactions between dietary components, and with other epigenetic regulators and specific genotypes [5,6,7,77,78]. Recent advances in two novel species, honey bees and locusts, are discussed in this section of the review because of their unique ability to provide insights into mechanisms underlying epigenetic regulation of phenotype and hence the propensity for disease. 

### 4.1. Genotype, Epigenotype and Phenotype

It has long been recognized that genotype does not necessarily determine phenotype, *i.e.*, observable traits or characteristics. In 1911, Johannsen suggested “Supposing that some organisms of identical genotypical constitution are developing under different external conditions, then these differences will produce more or less differences as to the personal qualities of the individual organisms” [79]. Environmental temperature, nutrition and interactions between these variables profoundly affect multiple aspects of mammalian development, morphology, physiology, behaviour, endocrinology, cell biology and molecular biology [80,81]. Indeed, the changes induced postnatally are so great that they could be mistakenly perceived as being due to genetic differences. Ultimately, these effects are mediated by epigenetic mechanisms, with numerous endogenous and exogenous regulators modifying gene expression via changes in epigenotype.

Phenotypic plasticity is common across species, and is the differential expression of alternative phenotypes from a single genotype depending on environmental conditions [82]. Changes occur at the level of organism, tissue or cell and this epigenetic remodelling allows individuals to respond and adapt to specific factors in the environment such as nutrition, temperature and social interactions (see Figure 2). In the nervous system, for example, epigenetic remodelling can significantly affect learning and memory. Marked structural and functional plasticity occurs in the brain during development and in adults. Whether epigenetic regulators are beneficial or harmful holds profound implications for phenotypic outcome in terms of brain function. Precise mechanisms underlying brain and neuronal plasticity are thus of considerable relevance and are providing new insights into mechanisms underlying neurological function. 

### 4.2. Nutrition, Honeybees and Epigenetics

Recent research suggests that the honeybee *Apis mellifera* may be an especially useful model for understanding the basis of learning, memory and cognition [83]. This species exhibits complex social, navigational and communication behaviours, and an impressive cognitive repertoire. These functions are undertaken by a brain containing only 1 million neurons compared with 100 billion in the human brain. Moreover, neural networks in the honeybee are limited in size and complexity, enabling the tracing of neural plasticity to specific neural circuits and single neurons.

A major focus of studies on nutritional regulation of epigenetic markers is DNA methylation. Level of food intake and multiple specific nutrients affect DNA methylation patterns in a diverse range of species [84,85,86,87,88]. In honeybee colonies, whether an individual becomes a queen or a worker depends not on genotype but on its diet during development. Female honeybee larvae develop into either a queen or worker phenotype, and determination of phenotype is due entirely to dietary-induced DNA methylation by royal jelly. This mixture of proteins, sugars and fatty acids, including 10-hydroxydecanoic acid and phenyl butyrate, reduces DNA methyltransferase 3 expression, leading to altered DNA methylation patterns that induce the queen bee phenotype [89].

Recent studies have shown that epigenetic mechanisms are important not only in development but also in behavioural changes during adult life [90]. Worker bees can be either nurses or foragers, depending on the needs of the hive for care of larvae versus collection of food and water. By contrast with the queen and worker castes, the phenotype of these subcastes is reversible. Whole genome bisulphite sequencing and comprehensive high-throughput array-based methylation analysis was used to compare methylomes in the distinct honeybee phenotypes. Especially important was the finding of reversible switching between epigenetic states in the two types of worker bee. Over 100 differentially methylated regions were found in nurse compared with forager bees, whereas there were no such differences in DNA methylation between the irreversible queen and worker castes. Similarly, there were marked differences in methylated regions when foragers were induced to revert to nursing. Regions of the genome involved in this response contained genes linked with development, ATP-binding, learning and axon migration, via transcriptional control and chromatin remodelling.

Current studies comparing long-lived queen and short-lived worker phenotype are proving useful for the study of diet-induced epigenetic effects on lifespan [91]. Further advances would undoubtedly result from investigation of the nutritional regulation of epigenotype in specific neurons and regions of the brain. These should provide novel insights into the role of nutrition in neurological development and function. 

### 4.3. Phenotypic Plasticity, Locusts and Neurological Function

One of the most dramatic forms of phenotypic plasticity occurs in locusts [82,92]. They can change reversibly between two extreme forms, and transformation between the solitarious form and the swarming gregarious form is driven by changes in population density and concomitant social interactions. These two forms of locust differ so extensively in appearance, morphology, physiology, neurochemistry and behaviour, that they were once thought to be two distinct species. For example, solitary locusts walk with a slow, creeping gait, fly mainly at night, have a restricted diet and avoid other locusts. By contrast, gregarious locusts have a rapid, upright gait, fly during the day, have a broad diet and are attracted to other locusts. 

The locust thus makes an outstanding model system in which to study epigenetic regulation of neurological phenotype. Brain size, structure and function are quite different in the two phenotypic forms of locust. The gregarious locust is smaller than the solitary form but its brain is some 30% larger. Moreover, marked differences in size of specific brain regions are related to function. Numerous differences also occur in individual neurons, neuronal connections and neurochemistry. Of considerable significance is the finding that serotonin (5 hydroxytryptamine, 5-HT), an evolutionarily conserved mediator of neuronal plasticity, is responsible for the extreme phenotypic transformation in the desert locust *Schistocerca gregaria* [82,92]. In the two phenotypic forms, marked differences occur in many neurotransmitters and neuromodulators, including dopamine, taurine, arginine, glycine and glutamate. However, it is serotonin alone that is both necessary and sufficient to induce the initial gregarious behaviour in locusts.

Genomic approaches have advanced understanding of many mechanistic aspects of phenotypic plasticity in locusts. Genome-wide gene expression profiling during switching between solitary and gregarious forms of the migratory locust *Locusta migratoria* has revealed changes in genes related to chemosensory proteins, the catecholamine pathway, and dopamine synthesis and synaptic release [93,94]. Very little is known about the specific epigenetic mechanisms involved in the phenotypic change of locusts. Studies in the migratory locust have, however, shown that it possesses genes that putatively encode methylation machinery, *i.e.*, DNA methyl transferases and a methyl-binding domain protein, and it exhibits genomic methylation, some of which appears to be localized to repetitive regions of the genome [95].

Taken together, these findings in locusts suggest that it could make an excellent model for investigating interactions between social and nutritional factors in regulating the epigenome and neurological phenotype.

## 5. Gene-Environment Interactions: The Serotonergic System and Brain Disorders

Elucidation of mechanisms underlying gene-environment interactions is central to an understanding of brain function and dysfunction. Many neurotransmitters and their associated pathways are implicated in brain disorders and their precise roles continue to be the subject of considerable discussion and controversy. To illustrate advances made in this field and the complexity of the multiple interacting processes involved, this section discusses gene-environment interactions in the serotonergic system, as a paradigm of the multiple key molecules and signalling pathways that are affected in brain disorders.

**Figure 4:**
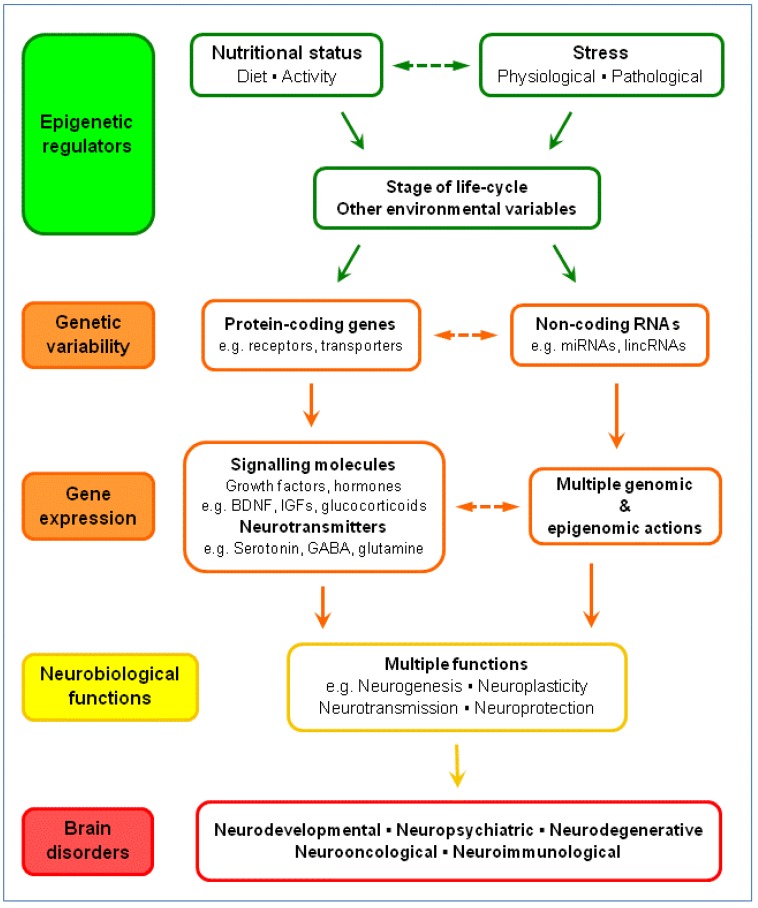
Nutrition-stress-gene interactions and brain disorders.

Serotonin is implicated in many forms of behavioural plasticity associated with social interactions. These range from marked behavioural differences in locusts to emotional well-being, depression and anxiety in humans [92]. Serotonin plays a key role in cognition and memory, and a dysfunctional serotonergic system is implicated in the pathophysiology of many neuropsychiatric and neurological disorders, including schizophrenia, Alzheimer’s disease and vascular dementia [96,97]. Recent progress in identifying mechanisms involved in these disorders suggests important roles for gene variants and epigenetic factors in modifying the serotonergic system. In addition, they suggest important interactions between the serotonergic system, other neurotransmitters including GABA and glutamine, and neuromodulators such as BDNF and glucocorticoids. An indication of the many critical interactions between genes, nutrition and stress in relation to neurological function and brain disorders is discussed in this section of the review and illustrated in Figure 4.

### 5.1. Dietary Tryptophan and Brain Serotonin

In mammals, the essential amino acid tryptophan is the precursor for serotonin and the extent to which nutrition interacts with the serotonergic system is therefore of considerable interest. Changes in brain tryptophan concentration rapidly influence the rate of neuronal serotonin synthesis. However, tryptophan is transported across the blood-brain barrier in competition with other large neutral amino acids (LNAA). Therefore serotonin levels do not necessarily reflect dietary tryptophan intake; instead there are complex interactions with the protein and amino acid content of the diet. This explains the impact of meals containing little or no protein on serotonin production. High-carbohydrate meals induce a striking difference in plasma tryptophan-LNAA acid ratios compared with high-protein meals, and may therefore affect brain tryptophan concentrations and serotonin synthesis [98]. Such meals also have distinct effects on the plasma tyrosine-LNAA ratio and hence have the potential to modify brain catecholamine synthesis. 

Serotonin synthesis from tryptophan occurs in presynaptic neurons and is vitamin B6 dependent [99]. After its release from synaptic vesicles, it acts via seven types of postsynaptic cell-membrane bound receptors (5-HTR) to modulate neurological function in multiple processes including cognition, learning, memory, mood and appetite. Actions of serotonin are terminated by its reuptake from the synaptic cleft into the presynaptic neurons. This occurs via a specific transmembrane transporter (SERT, 5-HTT). Key drugs for treatment of several neuropsychiatric disorders therefore target the serotonergic system, e.g., monoamine oxidase inhibitors prevent serotonin breakdown, and selective serotonin reuptake inhibitors (SSRIs) ensure that serotonin stays longer in the synaptic cleft.

Of particular interest in the present context is the finding that both SSRIs and herbal treatments increase BDNF in the hippocampus, a brain region critical for memory and cognition, while also reducing circulating glucocorticoids [100]. The aim was to compare a novel herbal treatment for anxiety disorder with conventional treatment using SSRIs. In a mouse model, the herbal treatment reduced anxiety-like behaviours, possibly via a reduction in circulating corticosterone and increase in BDNF levels in the hippocampus.

### 5.2. Brain Plasticity: BDNF, Glucocorticoids and Serotonin

Numerous growth factors, hormones and their specific receptors play a central role in mediating the effects of energy status on brain plasticity and function [5,6,7,8,64]. Especially relevant, therefore, are recent findings on the intimate cross-talk between glucocorticoids, BDNF and other trophic factors, especially in relation to dynamic brain plasticity [101,102,103].

Glucocorticoids and BDNF do not function alone as modulators of plasticity. Their effects are dependent on signals from many other neurotransmitters and intracellular molecules [101] Multiple lines of evidence suggest that BDNF plays a pivotal role in the pathophysiology of major depressive disorder, a common, chronic, recurrent mental illness that affects 10%–20% of the population [104]. Serum levels of mature BDNF, but not its precursor proBDNF, are decreased in patients with major depressive disorder [105]. Both BDNF and agonists of its receptor, TrkB, have antidepressant effects in animal models. This suggests a crucial role of the BDNF-TrkB signalling pathway in the therapeutic action of antidepressants such as SSRIs [104].

Neural plasticity in response to stress involves not only an increase in glucocorticoids but also requires numerous molecules including BDNF [101]. BDNF levels are highly dynamic in response to stress and vary across brain regions and over time. The transglutaminase 2 (TG2) inhibitor cysteamine is neuroprotective and increases TrkB signalling in the brain. Chronic administration of cysteamine ameliorates the decreases in TrkB in the frontal cortex and hippocampus and the anxiety/depression-like behaviours induced in mice by glucocorticoid treatment [106]. This indicates that BDNF-TrkB signalling plays an important role in the beneficial effects of cysteamine, suggesting that it would be a novel therapeutic drug for glucocorticoid-related symptoms of neuropsychiatric disorders. Moreover, TG2 may be a factor in the serotonin deficiency associated with major depressive disorder: increased levels of TG2 probably convert serotonin to Rac1, resulting in decreased levels of serotonin and BDNF that are associated with major depressive disorder [104].

### 5.3. Serotonergic System: Gene Variability, Epigenetics and Brain Disorders

Studies on the role of gene-environment interactions in neuropsychiatric disorders suggest that genetic and epigenetic changes in serotonin receptors (5-HTR) and transporter (5-HTT, SERT) are involved [107]. In peripheral blood leukocytes from patients with schizophrenia and bipolar disorder, DNA methylation in the promoter region of the serotonin type 1A gene (*5HTR1A*) is increased [108]. This would reduce gene expression and, if similar changes occur in the brain, would explain the decrease in receptor mRNA in these disorders. Support for this conclusion comes from the study of another serotonin receptor gene (*5HTR2A*): in post-mortem frontal brain samples from patients with schizophrenia and bipolar disorder compared with matched controls, genotype and DNA methylation interacted to fine-tune *5HTR2A* expression, and epigenetic down-regulation of *5HTR2A* was associated with early onset of disease [109].

Another focus of considerable interest and controversy in the field of gene-environment actions is the link between the serotonin transporter gene (*5HTT*, *SLC6A4*), stress and the *BDNF* gene in the risk of depression [110,111,112,113]. Recent evidence suggests a link between a functional deletion/insertion polymorphism, *5-HTTLPR*, in the serotonin transporter gene and environmental stresses over time, and especially during childhood and adolescence [114]. Adolescents homozygous for the short allele (SS) and exposed to early childhood adversities have a marked inability to process emotional information; a response linked with increased risk of depression and anxiety. Controversy exists in part because a description of the functional mechanisms involved has been lacking. However, findings on the functional anatomy of the serotonergic dorsal raphe nucleus suggest that genetic variation in serotonin uptake may affect the stress response and risk of depression through critical brain circuitry underlying stressor reactivity and regulation of emotion [115]. 

The precise role of the serotonergic system in brain disorders remains highly controversial. Nevertheless, these findings on genotype-epigenotype modifications of serotonin receptors and transporter suggest possibilities for novel therapeutic approaches based on the downstream effects of serotonin.

### 5.4. Nutrition-Stress-Gene Interactions and Brain Disorders

The present discussion of the serotonergic system’s role in brain disorders provides insight into the complexities, difficulties and potential benefits of elucidating the many signalling pathways and neural networks involved in gene-environment regulation of brain function. Multiple interactions occur between the serotonergic system and other neurotransmitter, neuromodulator and neurotrophic systems. Moreover, nutrition interacts with numerous environmental factors to determine overall impact on the epigenome of specific brain regions and cell types throughout the life-cycle. Stress is one such factor. Indeed, it is probable that individual differences in stress responses are modified by nutrition, and this in turn determines outcome in relation to the adverse effects of stress on multiple brain disorders [20,116]. 

Physiological stress can be defined as any external or internal condition that challenges the homeostasis of the cell or organism, and includes environmental stress, intrinsic development and aging [117]. The effects of the stress response can be beneficial or harmful, depending on the type and intensity of stress, its timing and duration, and individual differences in coping with stress [20,118]. Exposure to physiological and psychological stressors activates the hypothalamic-pituitary-adrenal axis (HPA), resulting in increased secretion of glucocorticoids. Moreover, variants of the glucocorticoid receptor gene (*NR3C1*) are linked with schizophrenia and bipolar disorder [119]. By contrast with its actions on glucocorticoids, stress down-regulates neurotrophins such as BDNF, thus contributing to the pathophysiology of brain disorders including depression, Alzheimer’s and Parkinson’s disease. Moreover, variants of the *BDNF* gene are linked with risk of neuropsychiatric disorders, including depression, eating disorders and schizophrenia [5,6,7,110,120].

Epigenetic mechanisms affect both short-term and long-term responses to stress and may even be inter-generational [121]. Compared with healthy controls, adult patients with psychosis have higher rates of childhood trauma, impairments in cognitive performance and smaller amygdala volume, a brain region important in emotional processing and higher functions such as working memory [122]. Considerable evidence suggests a key role for early-life nutrition, and especially prenatal maternal undernutrition, in risk of neurodevelopmental and neuropsychiatric disorders such as schizophrenia [5,6,7,123,124,125]. 

The central nervous system has a major role in metabolic control, and this is especially apparent in stressful events such as catabolic states and hormone deficiencies that mimic starvation [126]. Various nutrient, energetic and hormonal cues function in the hypothalamus to control glucose and lipid metabolism and overall energy balance. Hormones with nuclear receptors that act as transcription factors are especially potent mediators of nutrition-gene interactions [21,22]. Glucocorticoids, for example, mediate many of the effects of stress via epigenetic events involving DNA methylation, histone modifications and ncRNAs [127]. The growth arrest-specific 5 (Gas5) ncRNA is a riborepressor of the glucocorticoid receptor, and influences cell survival and metabolic activity during starvation by modulating the transcriptional activity of the receptor [128]. Moreover, the methylation states of many hormones and their receptors are modified by nutrition throughout the life-cycle. In humans, maternal intake of the methyl donor choline alters placental epigenomic marks and the epigenetic state of key modulators of placental and fetal HPA axis reactivity [129]. The possibility, therefore, is that maternal choline intake could be used to prevent the adverse effects of prenatal stress on subsequent neurological and neuropsychiatric disorders.

Deeper understanding of the genomic and epigenomic mechanisms underlying nutrition-stress interactions in the central nervous system should help to suggest novel approaches for prevention, treatment and alleviation of the multiple disorders associated with stress and sub-optimal nutrition, including cognitive impairment, dementia, depression, anxiety, schizophrenia, Alzheimer’s disease and Parkinson’s disease.

## 6. Conclusions

The current review has shown that knowledge of individual genetic variability and epigenetic regulation of gene expression underpins the understanding of nutritional regulation of optimal and sub-optimal brain health. Recent advances are providing insights into disorders ranging from cognitive impairment, depression and eating disorders to Alzheimer’s disease, schizophrenia and brain tumours. Future advances will involve very large-scale investigations of the whole genome and epigenomes of specific cell types, together with focused assessment of specific regulatory factors including protein-coding genes and non-coding RNAs (ncRNAs).

Considerable effort is now being made by large scale consortia to cope with the enormous sample sizes and vast amounts of data being generated by advances in genomics and epigenomics. The development of genome-wide association studies (GWAS) is enabling significant advances in identifying common disease-predisposing gene variants. However, the robust detection of disease-associated low-frequency alleles in complex diseases will require even larger study samples than those used in GWAS [36]. Moreover, studies combining GWAS with a systems genetics approach will undoubtedly advance understanding of the complex pathways linking genetics, environment and disease [37,38,130]. 

The International Human Epigenome Consortium (IHEC) incorporates inputs from scientists in North America, Europe, Australia, Japan and South Korea. Its primary goal is to provide free access to high-resolution reference human epigenome maps to the research community. A range of 21 normal and disease cell types are being investigated, including those from the fetal and adult nervous system. Epigenomic maps can be used to trace the origin of cells, dissect affected pathways and identify predictive biomarkers [46]. The hope is that these maps will have an impact on the understanding, treatment and management of many diseases. Underpinning these advances is the need for a global biobanking strategy to harmonize the multiple interests of organizations, initiatives, resources and stakeholders, including patients [131].

Technological advances cannot in themselves provide solutions to all problems associated with the aetiology and treatment of brain disorders. Future studies using innovative approaches and novel systems will also help to advance understanding of the links between nutrition and neuroscience. Some of these are highlighted in the present review. Evolutionary complexity is linked with increasingly large proportions of non-protein-coding sequences in the genome. Indeed, recent studies implicate ncRNAs in mediating changes in neural gene expression during evolution. They also suggest that the expansion of ncRNAs was in part responsible for the emergence of vertebrate complexity, especially in the brain, together with the increased cognitive and behavioural repertories of higher organisms [55]. Comparative genomic and epigenomic studies in a wide range of species will therefore be of particular benefit in elucidating mechanisms underlying brain disorders [86,132,133,134]. Key studies in development and neuroscience have been undertaken in many species including the fruit fly *Drosophila melanogaster*, zebra fish, frog and mouse. *Drosophila* has long been used as a model organism for studying diseases ranging from cancer to neurodegenerative disorders. Its versatile genetics and the ability to quickly generate multiple genetic variants could be of particular value in studying complex neuropsychiatric disorders such as autism and schizophrenia [134]. Moreover, studies in a range of species is currently enabling significant progress in understanding the role of the Wnt/β-catenin cell signalling pathway in many neurological and neuropsychiatric disorders, including Alzheimer’s, schizophrenia and the devastating brain cancer glioma [133]. 

In addition to the epigenetic studies in honeybees and locusts discussed in the current review, further novel approaches could, for example, involve molecular evolutionary studies of dietary selection. These should provide new insights into eating behaviours and disorders in humans. The giant panda *Ailuropoda melanoleuca* has a highly specialized bamboo diet that is quite different from the carnivorous or omnivorous diets of other bears. Comprehensive sequence analysis of the genes involved in the appetite-reward system suggests a complex genetic background, possibly involving miRNAs and deficiencies in dopamine metabolism, behind the panda’s dietary switch [135]. Progress in elucidating the complex role of nutrition in brain disorders will benefit considerably from future studies on ncRNAs. The predicted exponential rise in understanding of the role of ncRNAs in the central nervous system, combined with the versatility and relative simplicity of RNA chemistry, should make translation into diagnostic and therapeutic applications a reasonable possibility [56].

It is probable that genomic and epigenomic technologies will continue to advance significantly, resulting in reduced costs and increased opportunities. This should enable large numbers of individual genomes and epigenomes to be analysed throughout the life-cycle in relation to multiple environmental variables including specific diets, nutrients, energy intake and physical activity. In the long-term, outcomes should include major benefits in relation to optimization of life-style and ameliorating any underlying genetic propensity for disease. However, major cautions and caveats must also be mentioned. Current concern with respect to the ethical use of personal data will only increase as the functions of millions of newly identified gene variants are elucidated. Moreover, it is essential that nutritionists and dietitians are involved at all stages of such investigations. Significant advances are now being made in relation to two highly relevant areas: the accurate assessment of dietary intake and life-style, and the education of professional nutritionists in the molecular and cellular basis of health and disease. 

In summary, future advances in genomics and epigenomics will continue to provide new insights into the mechanisms that underpin the nutritional regulation of gene expression in the brain. Technological progress cannot in itself provide solutions to all biomedical problems. Innovative approaches combined with state-of-the-art techniques should suggest possibilities for preventing, ameliorating and treating the multiple complex brain disorders associated with adverse genetic and environmental factors.
